# The Role of Sauna Bathing in Ischemic Heart Disease: A Narrative Review of Therapeutic Potential, Physiological Mechanisms, and Emerging Clinical Applications

**DOI:** 10.7759/cureus.98162

**Published:** 2025-11-30

**Authors:** Georges Hachem, Jad Rafic Slim, Bashir Imam, Usama Hassan Nawaz, Maha Razzaq, Mohamed Touny, Fnu Hafsa, Manoj Argariya, Syam P Maharaj, Sara Mueen, Kazuaki Sato

**Affiliations:** 1 Faculty of Medicine, American University of Beirut, Beirut, LBN; 2 Faculty of Medicine, University of Balamand, Beirut, LBN; 3 Pediatrics, University of Pittsburgh Medical Center, Coudersport, USA; 4 Neurorehabilitation, Queen Mary’s Hospital, London, GBR; 5 Internal Medicine, Rashid Latif Medical College, Lahore, PAK; 6 Cardiology, National Heart Institute, Giza, EGY; 7 General Physician, Shadan Institute of Medical Sciences, Hyderabad , IND; 8 General Internal Medicine, Royal Blackburn Hospital (East Lancashire Hospital Trust), Blackburn, GBR; 9 Critical Care, San Fernando General hospital, San Fernando, TTO; 10 Cardiology, Dow International Medical College, Karachi, PAK; 11 Emergency Medicine, Yokota Air Base Hospital (374th Medical Group), Fussa, JPN

**Keywords:** exercise therapy, ischemic heart disease, sauna bathing, steam bath, vasodilation

## Abstract

Ischemic heart disease (IHD) continues to be one of the main causes of morbidity and mortality among the general adult population. It occurs due to a lack of sufficient coronary blood flow. Sauna bathing has recently been reported to give cardiovascular benefits that are similar to those from a moderate level of exercise. The proposed benefits include increased vascular function, lower blood pressure, and improved cardiac performance. Thus, sauna therapy could be considered as an adjunctive intervention for patients with IHD. This review assesses the existing clinical research on the therapeutic use of sauna bathing in managing IHD, the physiological pathways contributing to its protective effects, and future research directions. Relevant studies were identified in PubMed and Google Scholar (2015-2025) using keywords such as IHD, sauna bathing, and steam bath. The studies showed that with consistent usage of Finnish or far-infrared saunas, patients with IHD gain better hemodynamic stability, endothelial function, autonomic balance, and anti-inflammatory activity. Observational studies have been most consistent in showing that sauna use produces a favorable impact on the cardiovascular system. However, the results of randomized controlled trials offer rather mixed conclusions. In conclusion, while sauna treatment seems safe and beneficial (especially for patients who are not able to undergo regular exercise), it warrants the need for unitary protocols and larger, longer-lasting trials to both establish its effectiveness and clarify its position in cardiovascular prevention and rehabilitation.

## Introduction and background

Ischemic heart disease (IHD), also known as coronary artery disease (CAD), is characterized by the progressive and cumulative buildup of fatty deposits called atherosclerotic plaques within the coronary arteries, which leads to significant narrowing of the arterial lumen and consequently results in a substantial reduction of blood flow and oxygen delivery to the myocardium. This impairment in myocardial perfusion is the underlying pathophysiological basis for chest pain (angina), myocardial infarction, and several other ischemic complications that collectively account for the majority of deaths due to heart-related diseases worldwide [1,2]. Despite remarkable advancements in diagnostic imaging, interventional cardiology, and pharmacologic therapies, IHD continues to represent a persistent and evolving public health challenge, affecting millions of individuals globally and placing a tremendous and escalating strain on healthcare systems, economic productivity, and clinical resources. Growing evidence suggests that beyond traditional medical and surgical interventions, novel and adjunct lifestyle-based strategies may play a significant role in reducing this global burden. As a result, there has been increasing attention toward non-pharmacologic approaches that can not only support cardiovascular health but also improve vascular tone, enhance the ability of blood vessels to function properly, and regulate autonomic nervous system activity [3]. These complementary therapeutic strategies offer the potential to enhance traditional medical management while minimizing adverse effects and healthcare costs. Sauna bathing has been a part of Finnish culture for centuries, and now it's seen as a safe and helpful way to support cardiovascular health and overall well-being [4]. The longstanding tradition of sauna use in Nordic countries provides valuable observational data spanning multiple generations, offering unique insights into long-term health outcomes associated with regular thermal therapy. Sauna bathing involves exposing the body to elevated temperatures in an environment that is typically dry. The practice has been around for millennia, and it involves heat at around 70-100 °C, produced by pouring water over hot stones [5]. This ancient therapeutic modality has transcended cultural boundaries and is now practiced worldwide, with various adaptations and modifications to suit different populations and preferences. There are several common sauna modalities, including traditional Finnish dry saunas, infrared saunas, and Waon therapy [6]. Each of these approaches offers distinct characteristics in terms of heat delivery mechanisms, temperature ranges, and physiological responses, allowing for personalized selection based on individual tolerance and therapeutic goals. Sauna bathing induces a controlled thermal stress that triggers several important and clinically relevant cardiovascular responses: increased heart rate and cardiac output, vasodilation, reduced systemic vascular resistance, and a temporary drop in blood pressure during the recovery phase [7]. These hemodynamic changes closely resemble those observed during moderate-intensity physical exercise, suggesting shared mechanistic pathways and potential therapeutic overlap. Repeated sauna use may mimic some of the cardiovascular benefits of moderate aerobic exercise, without requiring physical exertion, making it a promising modality for older adults or those with physical limitations such as musculoskeletal disorders, severe deconditioning, or mobility impairments [3]. This accessibility represents a significant advantage for populations who may be unable or unwilling to engage in conventional exercise-based cardiac rehabilitation programs. Frequent sauna use has been linked to a lower risk of sudden cardiac death, fatal coronary events, deaths related to cardiovascular disease, and overall mortality, demonstrating a dose-response relationship with greater frequency of use associated with more pronounced protective effects [8]. However, sauna breathing itself has some safety considerations as well. Consumption of alcohol before sauna therapy can cause hypotension and arrhythmias [3]. Additionally, previous myocardial infarction and severe aortic stenosis are contraindications of sauna therapy due to increased oxygen demand [3]. This review aims to explore the physiological mechanisms and clinical benefits of sauna bathing in the context of IHD, to inform evidence-based integration into cardiovascular care and guide future research. It will first assess the existing clinical research on sauna bathing in managing IHD, then explore the physiological pathways contributing to its protective effects, and finally discuss emerging therapeutic applications and future research directions.

## Review

Methodology

This narrative review explores sauna bathing's therapeutic potential and mechanisms in IHD. A literature search was conducted on PubMed and Google Scholar (inception to June 2025, English only). Keywords included "Sauna bathing," "steam bath," and "ischemic heart disease”. Human studies and relevant review articles on cardiovascular outcomes and mechanisms, including Finnish and infrared saunas, were included. Animal studies, non-English works, and non-cardiovascular-focused studies were excluded. This is a narrative review and involves a qualitative synthesis of the literature published online since inception to July 2025. The literature search has been summarized in Table 1.

**Table 1:** Results of literature search.

Search	Query (Keyword)	Results
1	(Ischemic heart disease)	599,308
2	(Steam bath)	954
3	(Sauna Bathing)	431
4	(Ischemic heart disease) AND (Steam bath)	54
5	(Ischemic Heart Disease) AND (Sauna Bathing)	29
6	((Steam bath) OR (sauna bathing)) AND (Ischemic heart disease)	57

Therapeutic potential of sauna bathing in IHD 

Clinical Evidence from Finnish Sauna Studies

The strongest evidence supporting the use of sauna bathing as a cardioprotective intervention comes from long-term observational studies conducted in Finland, where sauna use is a part of cultural tradition. The most well-known of these is the landmark of Kuopio Ischemic Heart Disease Risk Factor Study (KIHD), which followed over 2,300 middle-aged Finnish men for more than 20 years. The study found a remarkably strong and graded inverse relationship between sauna frequency and cardiovascular mortality. Compared to those who bathed once weekly, men who used a sauna two to three times per week had a statistically significant 22% lower risk of sudden cardiac death (SCD) [5]. Those using it four to seven times per week experienced a 63% reduction in SCD and a 50% reduction in fatal cardiovascular disease (CVD) [9]. Session length also mattered; sessions longer than 19 minutes were associated with approximately half the SCD risk compared to shorter sessions under 11 minutes [8].

These effects were observed even after adjusting for independent of traditional cardiovascular risk factors, suggesting that sauna bathing itself may have a protective influence. Short-term effects have also been documented. In a 2017 experimental study, a single 30-minute Finnish sauna session led to an acute drop in systolic blood pressure from 137 to 130 mmHg and diastolic from 82 to 75 mmHg. Arterial stiffness, measured by carotid-femoral pulse wave velocity (cf-PWV), also declined significantly from 9.8 to 8.6 m/s immediately post-sauna [5].

Beyond hemodynamic changes, frequent sauna use may help regulate inflammation. In a 2018 cohort analysis, frequent users (four to seven sessions per week) had lower levels of high-sensitivity C-reactive protein (hsCRP) and reduced leukocyte counts, both markers of systemic inflammation [9]. However, shorter-term randomized trials show more mixed results. A 2023 eight-week randomized controlled trial (RCT) of Finnish sauna use in patients with stable coronary artery disease (four sessions per week, 20-30 minutes per session) found no significant improvement in endothelial function, microvascular health, arterial stiffness, or blood pressure, despite signs of heat acclimation [10]. This contrast between large-scale observational outcomes and short-term RCTs may reflect differences in exposure duration. Sauna’s benefits likely accumulate gradually, through long-term physiological changes or indirect pathways like stress reduction and improved sleep, which might not be captured by standard vascular markers. It’s also possible that healthier lifestyle patterns among sauna users partly contribute to the observed outcomes [8,9]. Moreover, the therapeutic window for sauna benefits appears to extend beyond traditional cardiovascular markers. Recent investigations have explored the impact of sauna bathing on metabolic parameters, including lipid profiles and glucose metabolism, which are intimately linked to IHD progression. A 2019 study by Kunutsor et al. demonstrated that frequent sauna users exhibited significantly lower levels of total cholesterol and LDL cholesterol compared to infrequent users, independent of other lifestyle factors [11].

Clinical Evidence From Infrared Sauna Studies

Far-infrared sauna (FIRS) therapy is a form of passive heat treatment that uses infrared radiation to warm the body directly, allowing for deeper tissue penetration than traditional saunas. This deeper heat triggers vigorous sweating and cardiovascular activity at lower ambient temperatures, making it more tolerable for individuals with exercise limitations. The cardiovascular effects of FIRS resemble those of moderate exercise. Heat exposure leads to vasodilation, sweating, reduced afterload, increased heart rate, and elevated cardiac output, mimicking the physiological demands of brisk walking. As a result, FIRS can serve as an alternative for individuals with mobility issues, arthritis, CVD, or respiratory limitations, who may find traditional exercise challenging. Though the evidence base is smaller than for Finnish saunas, FIRS has shown particular promise in managing chronic heart failure (CHF). The study reported significant improvements in endothelial function, cardiac performance, and clinical symptoms like dyspnea, fatigue, and edema following repeated FIRS use [12]. The therapy also led to reductions in B-type natriuretic peptide (BNP), a biomarker of heart failure severity, and increases in ejection fraction and walking distance [12]. Similarly, Miyamoto et al. reported significant improvements in ejection fraction, walking distance, and reduced catecholamine levels in CHF patients following FIRS therapy. These findings underscore the potential for FIRS to support not only hemodynamic stability but also functional capacity and quality of life in vulnerable patient populations [13]. While some manufacturers claim that FIRS can normalize blood pressure, lower cholesterol, and reduce stress, the evidence remains modest, and larger trials are needed to validate these claims for individuals with coronary risk factors. Nonetheless, the distinct mechanism of infrared heat penetration, combined with its gentler thermal profile, points to FIRS as a valuable alternative for patients who may not tolerate high-heat environments. This broader applicability enhances the role of heat therapy in preventive cardiology and rehabilitation, particularly for those with mobility limitations or sensitivity to conventional exercise regimens. These findings stress the need to differentiate between sauna types when assessing therapeutic potential. Future studies should further explore how variations in heat delivery methods influence cardiovascular outcomes, helping tailor sauna-based therapies to specific patient populations (Table 2). 

**Table 2:** Summary of key clinical outcomes from sauna bathing studies in IHD/CVD. SCD, sudden cardiac death; CHD, coronary heart disease; CVD, cardiovascular disease; cf-PWV, carotid-femoral pulse wave velocity; SBP, systolic blood pressure; DBP, diastolic blood pressure; hsCRP, high-sensitivity C-reactive protein; FMD, flow-mediated dilation; BP, blood pressure; BNP, B-type natriuretic peptide; IHD, ischemic heart disease

Study (author, year)	Sauna type	Population/condition	Study design	Key outcomes measured	Key findings/effect
Laukkanen et al., 2015 (KIHD) [3]	Finnish	Middle-aged Finnish men (*n* = 2,315)	Observational cohort (20.7 years follow-up)	Sudden cardiac death (SCD), fatal CHD, fatal CVD, all-cause mortality	-Reduced risk of SCD (22% for 2-3x/week, 63% for 4-7x/week). Reduced fatal CVD (50% for 4-7x/week); Sessions >19 minutes halved SCD risk. Benefits persisted after CVD risk factor adjustment.
Laukkanen et al., 2017 [5]	Finnish	Healthy subjects	Experimental	Systolic/diastolic blood pressure, cf-PWV	Acute reduction in SBP (137-130 mmHg) and DBP (82-75 mmHg); reduced cf-PWV (9.8-8.6 m/s) immediately post-sauna.
Kunutsor et al., 2018 [9]	Finnish	Middle-aged Finnish men	Observational cohort	Inflammatory markers (hsCRP, fibrinogen, leukocytes)	Frequent sauna (4-7x/week) is associated with lower hsCRP and leukocyte count.
Debray et al., 2023 [10]	Finnish	Adults with stable CAD (*n* = 41)	Randomized controlled trial (8 weeks)	Endothelial function (FMD), microvascular function, central arterial stiffness (cf-PWV), and blood pressure	No significant improvement in FMD, microvascular function, cf-PWV, or BP over 8 weeks. Evidence of heat acclimation
Kihara et al., 2002 [11]	Far-infrared	Patients with chronic heart failure (CHF)	Clinical studies	endothelial function, cardiac function, hemodynamic variables, clinical symptoms (dyspnea, fatigue, edema), BNP levels, ejection fraction, walking distance	Improved peripheral vascular endothelial function, improved cardiac function, improved clinical symptoms, decreased BNP, improved ejection fraction, and increased walking distance.
Miyamoto et al., 2005 [12]	Far-infrared	Patients with CHF	Clinical study	CHF symptoms, ejection fraction, walking distance, catecholamine levels	Improved CHF symptoms, ejection fraction, and walking distance; decreased catecholamine levels.

Physiological mechanisms underlying sauna’s cardiovascular effect in IHD

Thermal Effects on Cardiovascular Physiology and Hemodynamics in IHD

According to findings by Poikonen et al., sauna bathing leads to a notable increase in core body temperature, which typically rises by 0.4 to 2 °C, depending on the duration of exposure, humidity level, and temperature of the sauna [7]. This thermal stress activates the body’s homeostatic thermoregulatory mechanisms designed to dissipate excess heat and restore temperature balance. The primary physiological responses include evaporative cooling through sweating, cutaneous vasodilation, and a compensatory increase in cardiac output to facilitate efficient heat transfer to the skin surface. A key adaptation during sauna bathing is the marked vasodilation of skin blood vessels, which significantly redistributes blood flow. Normally, the skin receives only 5%-10% of total cardiac output, but during sauna exposure, this can surge to nearly 70% (approximately 7-8 L/minute) [14]. This massive increase in peripheral circulation promotes heat dissipation but concurrently reduces systemic vascular resistance, leading to a transient drop in arterial blood pressure. To counterbalance this, cardiac output and heart rate increase, ensuring adequate perfusion to vital organs. These hemodynamic responses closely resemble those observed during moderate-intensity physical exercise, where heart rate, stroke volume, and cardiac output all rise to meet increased metabolic demands. Consequently, sauna bathing can be viewed as a form of passive cardiovascular conditioning, offering effects that parallel exercise-induced adaptations. Accumulating evidence indicates that both regular physical activity and periodic elevation of heart rate serve as robust predictors of improved cardiovascular health and reduced all-cause mortality [15].

Thus, Sauna bathing offers cardiovascular benefits similar to exercise without musculoskeletal strain, making it ideal for patients unable to train. It serves as a supportive tool in cardiac rehabilitation and preventive care [16].

Impact on Endothelial Function and Nitric Oxide Bioavailability

Endothelial dysfunction is a key early feature of IHD, preceding atherosclerotic plaque formation and contributing to impaired vascular tone, inflammation, and thrombosis [17]. Sauna therapy has demonstrated consistent improvement in endothelial function, with Sobajima et al. reporting a 1.8% increase in brachial artery flow-mediated dilation (FMD) in patients with IHD after regular sauna use [18]. While NO levels were not directly measured, this improvement likely reflects increased nitric oxide (NO) bioavailability. Supporting this, Brunt et al. showed that thermal therapy enhances FMD through NO-dependent mechanisms [19]. NO, synthesized by endothelial nitric oxide synthase (eNOS), plays a central role in vascular homeostasis, promoting vasodilation, reducing platelet aggregation, and inhibiting leukocyte adhesion [20]. Sauna-induced heat stress increases heart rate, cardiac output, and peripheral blood flow, elevating shear stress on the endothelium. This shear stress stimulates NO release, enhancing vascular function. Kihara et al. reported a 68% rise in brachial artery blood flow, supporting this mechanism [21]. This rise is a known stimulus of eNOS; therefore, production of NO was promoted, which in turn contributed to the vasodilatory and endothelial repair effects of sauna therapy [22]. Repeated thermal exposure has also been shown to upregulate eNOS expression, with Brunt et al. reporting a 1.4-fold increase in serum eNOS protein concentrations [19]. Given that endothelial dysfunction underlies IHD progression, sauna therapy may offer disease-modifying potential. Similarly, Comini et al. showed that ACE inhibitors enhance eNOS activity, increasing NO availability. This shared mechanism suggests sauna therapy could complement conventional cardiovascular treatments, offering a synergistic approach to improving vascular health [23]. The temporal dynamics of endothelial adaptation to repeated sauna exposure warrant particular attention. While acute sauna sessions produce immediate improvements in endothelial function lasting several hours post-exposure, chronic regular use appears to induce sustained baseline improvements in vascular health. This pattern suggests both acute stimulus-response mechanisms and longer-term vascular remodeling processes. The cumulative effect of repeated thermal stress may promote structural changes in the arterial wall, including modifications in extracellular matrix composition and smooth muscle cell phenotype, contributing to enhanced vascular compliance and reduced atherosclerotic vulnerability [24].

Modulation of the Autonomic Nervous System

Sauna bathing triggers a well-characterized biphasic autonomic response, initiated with immediate sympathetic nervous system activation upon heat exposure, followed by substantially increased parasympathetic activity to maintain physiological homeostasis. The intense heat activates the sympathetic system, causing marked peripheral vasodilation to significantly enhance skin blood flow and efficiently dissipate heat from the body's core. During the post-sauna recovery period, parasympathetic activity becomes progressively dominant, leading to a profound calming effect on the cardiovascular system, as evidenced by a significant heart rate reduction (average drop from 77 to 68 beats per minute in a study of 93 people, indicating substantially improved autonomic balance) [25]. This recovery phase shows markedly increased heart rate variability (HRV), indicating improved autonomic function and cardiovascular flexibility, which Jarczok et al. linked to a significantly lower mortality risk [26]. Sauna bathing mimics the train-and-recover pattern of structured interval training, providing comparable cardiovascular advantages for cardiovascular health through passive thermal stress. Brunt et al. reported clinically meaningful reduced arterial stiffness (5%-10%) and a substantial 6 mmHg drop in systolic blood pressure [19]. Therefore, sauna bathing replicates the beneficial vascular effects of aerobic exercise, serving as a highly effective passive workout that is particularly beneficial for patients with autonomic imbalance, such as those with IHD [12]. Thus, sauna offers a valuable and accessible non-pharmacological way to enhance cardiovascular regulation and reduce overall disease risk in vulnerable populations.

Arterial Stiffness and Blood Pressure Regulation

Sauna bathing has been consistently shown to exert significant and clinically meaningful beneficial effects on arterial stiffness and blood pressure, both of which are essential parameters in maintaining optimal cardiovascular health [27,28,29]. Vasodilation from heat exposure, alongside markedly improved endothelial function, lowers systemic blood pressure through multiple interconnected mechanisms. Research shows that regular sauna use decreases both systolic and diastolic readings in a dose-dependent manner. For instance, in one well-controlled experimental investigation, a single 30-minute session acutely reduced blood pressure from 137/82 to 130/75 mmHg, with systolic pressure remaining substantially lower during the post-sauna recovery period [5,30]. Beyond the immediate blood pressure reduction after a session, consistent and repeated sauna bathing offers significant long-term benefits of sustained blood pressure improvement that persist even on non-sauna days [31]. This lasting effect is thought to be due to the progressive strengthening of the endothelium, which improves its ability to dilate and regulate blood flow effectively over time [3]. Additionally, sauna bathing improves arterial compliance, leading to a measurable reduction in arterial stiffness. Studies have shown that frequent sauna exposure is linked to a significant decrease in cf-PWV, a key quantitative indicator of better vascular flexibility and reduced cardiovascular risk [5]. The combined effects of lower blood pressure and enhanced arterial compliance synergistically reduce the heart's workload by decreasing overall vascular resistance. These beneficial factors discussed earlier are well-established and known to improve clinical outcomes in patients with IHD [32,33]. This reduced cardiac demand is especially beneficial for those with IHD by effectively supporting myocardial function and substantially lessening the hemodynamic strain on their compromised cardiovascular system. The sustained blood pressure reductions observed with regular sauna bathing may be partially mediated through favorable modulation of autonomic nervous system activity. Studies demonstrate that sauna exposure in habituated individuals primarily elevates noradrenaline rather than a full catecholamine response, suggesting a more refined stress adaptation [34]. The post-sauna recovery period is characterized by enhanced parasympathetic reactivation and reduced sympathetic tone, contributing to prolonged blood pressure-lowering effects that extend beyond the immediate post-exposure period [25]. Furthermore, the arterial compliance improvements documented following sauna exposure appear dose-dependent, with frequent users demonstrating more pronounced reductions in arterial stiffness and enhanced vascular flexibility compared to infrequent users [30].

Inflammatory and Oxidative Stress Pathways

Chronic low-grade inflammation and oxidative stress are well-established contributors to the initiation and progression of atherosclerosis and IHD [35,36]. Emerging evidence suggests that regular sauna bathing may exert cardioprotective effects by modulating these pathological pathways, potentially reducing both inflammatory burden and oxidative damage [37,38]. A study on Finnish sauna bathing demonstrated that men who engaged in sauna bathing four to seven times per week had significantly lower levels of hsCRP, a key marker of inflammation, as well as reduced leukocyte counts, compared to those who used the sauna only once per week [37]. This inverse relationship between sauna frequency and circulating inflammatory markers, such as hsCRP and leukocyte count, suggests that reduced systemic inflammation may serve as a key mechanistic link between frequent sauna use and the observed lower risk of both acute and chronic diseases. It is important to note here that acute sauna exposure may transiently elevate certain inflammatory cytokines, such as interleukin-6 (IL-6) and interleukin-1 receptor antagonist (IL-1RA) [39]. However, with consistent sauna use, the cumulative effect appears to be anti-inflammatory, as evidenced by reductions in baseline levels of systemic inflammatory markers [37]. This biphasic response - characterized by an acute, transient increase in inflammatory cytokines followed by long-term reduction, could be an adaptive process that boosts the body's resilience to chronic inflammation and contributes to immune regulation over time. Oxidative stress, caused by excess reactive oxygen species (ROS), leads to endothelial dysfunction and arterial damage, promoting IHD [40,41]. Heat therapy offers protection by enhancing the body’s resistance to oxidative stress [38,42,43]. Regular sauna bathing has also been associated with reductions in oxidative stress, further contributing to its protective properties in IHD [43].

Emerging therapeutic applications and clinical integration

Synergistic Effects With Exercise Training

The integration of sauna bathing with conventional exercise-based cardiac rehabilitation represents a promising therapeutic strategy that capitalizes on complementary physiological mechanisms. A landmark RCT by Lee et al. provided compelling evidence for the synergistic benefits of combining post-exercise sauna therapy with standard exercise training in adults with CVD risk factors [44]. This eight-week intervention study randomized 47 participants aged 49 ± 9 years with low physical activity levels and at least one traditional CVD risk factor into three groups: guideline-based regular exercise with 15-minute post-exercise sauna (EXS), guideline-based regular exercise alone (EXE), or control (CON).

The combined intervention group demonstrated superior improvements compared to exercise alone across multiple cardiovascular parameters [44]. When compared to exercise alone, the EXS group displayed significantly greater improvements in cardiorespiratory fitness (+2.7 mL/kg/min; 95% confidence interval (CI), +0.2 to +5.3 mL/kg/min), lower systolic blood pressure (-8.0 mmHg; 95% CI, -14.6 to -1.4 mmHg), and reduced total cholesterol levels [44]. These findings suggest that sauna bathing substantially supplements the cardiovascular benefits achieved through exercise alone.

The mechanistic underpinnings of these synergistic effects reflect the complementary nature of exercise-induced and thermal stress responses. Exercise training primarily induces cardiovascular adaptations through mechanical shear stress on the vascular endothelium and metabolic demands that stimulate mitochondrial biogenesis and oxidative capacity [45]. Conversely, passive heat therapy generates distinct thermal stress that activates multiple cardiovascular protective pathways. A study by Brunt et al. demonstrated that 8 weeks of passive heat therapy via hot water immersion improved endothelium-dependent dilation, reduced arterial stiffness, decreased carotid intima-media thickness, and lowered blood pressure in sedentary adults, indicating comprehensive improvements in cardiovascular health [19]. These thermal-induced adaptations appear to occur through enhanced NO bioavailability, improved autonomic balance, and modulation of inflammatory pathways [19].

Heat shock proteins (HSPs), particularly HSP70, play a crucial role in mediating the cardiovascular protective effects of thermal stress. HSP70 is a chaperone protein induced by various cellular stresses and has been recognized for its involvement in preventing atherosclerotic CVD [46]. These proteins confer cytoprotective effects through enhanced protein folding, reduced oxidative stress, and modulation of inflammatory signaling cascades [47]. The combination of exercise-induced mechanical stress and sauna-induced thermal stress appears to produce amplified cardiovascular benefits that exceed those achievable through either modality alone.

From a clinical rehabilitation perspective, post-exercise sauna bathing offers pragmatic advantages for cardiac rehabilitation programs. A prospective study by Ohori et al. examining the combined effects of repeated sauna therapy and exercise training in patients with CHF demonstrated that the addition of exercise training programs to repeated sauna therapy was efficient and effective for improving cardiac function and daily activities [48]. Critically, in the Lee et al. study, the addition of 15-minute sauna sessions did not require substantial extension of rehabilitation session duration, as sauna exposure could be integrated into the post-exercise cool-down period [44]. This efficiency makes combined sauna-exercise protocols particularly attractive for implementation in resource-constrained rehabilitation settings where maximizing outcomes per unit of patient engagement time is paramount.

Precision Medicine Approaches

The emerging paradigm of precision medicine recognizes substantial inter-individual variability in therapeutic responses, and sauna therapy represents no exception to this principle. Accumulating evidence from large-scale prospective cohort studies suggests that individual responses to thermal therapy vary considerably based on genetic, physiological, and clinical factors, necessitating stratified approaches to optimize therapeutic efficacy while minimizing potential risks in vulnerable populations [8].

Genetic polymorphisms affecting key physiological pathways represent important determinants of sauna responsiveness, though this area remains relatively understudied. HSP gene expression, particularly variations in the HSP70 family (HSPA1A and HSPA1B loci), theoretically influences individual responses to thermal stress, given the central role these proteins play in cardiovascular protection [46]. Similarly, polymorphisms in eNOS genes may modulate the magnitude of vasodilatory responses to heat stress, as NO bioavailability is a key mediator of heat therapy's cardiovascular benefits [19]. However, prospective studies specifically examining genetic predictors of sauna therapy response in CVD populations are needed to translate these mechanistic insights into clinical practice.

Beyond genetic factors, baseline clinical characteristics substantially modify sauna therapy responses and safety profiles. Patients with diabetes mellitus warrant particular consideration, as impaired thermoregulatory mechanisms and increased susceptibility to dehydration may compromise both safety and efficacy [49]. Diabetic autonomic neuropathy further complicates cardiovascular responses to thermal stress, potentially blunting adaptive heart rate responses and increasing orthostatic intolerance risk [50]. The presence of CHF, while not an absolute contraindication, requires careful monitoring and individualized protocols, as demonstrated in studies of Waon therapy (a form of sauna bathing at 60 °C) that have shown both safety and efficacy when appropriately implemented [48].

Concomitant medications represent another critical modifier of sauna therapy response and safety. Antihypertensive agents, particularly diuretics, beta-blockers, and vasodilators, interact with thermal stress to influence hemodynamic stability [51]. Diuretic therapy may increase dehydration risk during sauna exposure, while beta-blockers can blunt compensatory heart rate responses to heat-induced vasodilation. These medication effects necessitate individualized protocol adjustments and enhanced monitoring strategies.

The integration of these multiple modifying factors into evidence-based clinical decision algorithms remains an underdeveloped area requiring urgent attention. Current guidelines lack specificity regarding patient selection criteria, contraindication thresholds, and individualized dosing parameters for sauna therapy in CVD management [3]. Development of validated risk stratification tools that incorporate genetic markers, comorbidity profiles, medication regimens, and baseline functional capacity represents a critical priority for advancing sauna therapy from population-level recommendations to truly personalized cardiovascular interventions. Such stratification would enable clinicians to identify patients most likely to benefit from thermal therapy while minimizing risks in vulnerable subpopulations.

Integration With Digital Health Technologies

The convergence of thermal therapy with digital health technologies offers unprecedented opportunities to enhance safety monitoring, optimize therapeutic protocols, and facilitate widespread implementation of sauna-based cardiovascular interventions. Contemporary wearable biosensors capable of continuous monitoring of heart rate, blood pressure, and peripheral temperature provide real-time physiological surveillance during sauna exposure, enabling immediate detection of adverse responses and personalized adjustment of session parameters [52]. Advanced wearable devices incorporating multiparameter algorithms can calculate individualized thermal dose exposure and provide alerts when physiological thresholds are approached, substantially enhancing safety profiles for home-based therapy.

Smart sauna systems equipped with automated environmental controls represent another technological advancement, facilitating precision thermal dosing. These systems can integrate user-specific profiles with real-time physiological feedback to dynamically adjust temperature, humidity, and session duration, ensuring optimal therapeutic stimulus while maintaining safety margins. While sophisticated integrated systems remain in development, the fundamental technology for remote physiological monitoring and automated environmental control exists and has been successfully implemented in related therapeutic contexts [53].

Mobile health applications extend the capabilities of technology-enabled sauna therapy through comprehensive session tracking, symptom logging, and patient education modules. Such applications can facilitate integration with electronic health records, enabling seamless communication between patients and healthcare providers and facilitating timely intervention when concerning patterns emerge [54]. Telemedicine platforms allow for remote consultation and protocol adjustments without requiring in-person visits, dramatically expanding access to specialized guidance for patients in geographically remote or underserved areas [55].

The feasibility of home-based thermal therapy with remote monitoring has been demonstrated in related contexts. Studies of passive heat therapy via hot water immersion have successfully implemented standardized protocols with regular remote follow-up, demonstrating excellent adherence and safety profiles [19]. While infrared sauna therapy differs from traditional Finnish sauna in temperature and humidity characteristics, the principles of remote monitoring and standardized protocols remain applicable across thermal therapy modalities [19].

Safety and contraindications

Sauna bathing offers numerous cardiovascular benefits, such as reduced arterial stiffness, lower blood pressure, and improved lipid profiles and left ventricular ejection fraction [3]. However, certain cardiac conditions necessitate considerable caution. Patients with unstable angina should strictly avoid sauna use due to the potential for increased myocardial oxygen demand. Similarly, recent myocardial infarction and severe aortic stenosis are absolute contraindications unless specifically cleared by a cardiologist [27]. Consuming alcohol before or during sauna sessions should be strictly avoided, as it significantly heightens the risk of low blood pressure, dangerous heart rhythm disturbances, and SCD [3]. According to a comprehensive study in Finland, around 102 sudden deaths occurred within 24 hours following the use of sauna, in which approximately 30% seemed to be directly linked to alcohol related accidents [31]. Initial sauna sessions for first-time users should be relatively brief (10-15 minutes) and gradually increased, not exceeding 45 minutes [56]. Elderly individuals with orthostatic hypotension should carefully avoid sudden temperature changes [57], and pregnant women should always seek medical advice before use [58]. While fatal events linked to sauna use are extremely rare and often associated with alcohol, adhering to appropriate safety guidelines allows individuals to potentially enjoy its health benefits while minimizing risks.

Limitation of existing studies

While sauna therapy has demonstrated considerable and significant potential benefit in the treatment of IHD, a significant limitation in the studies still exists. Since this is a narrative review, formal statistical analysis, including but not limited to a meta-analysis, was not performed, and this could affect the conclusions derived from the paper. The reduced risk of IHD with Finnish sauna use may also be influenced by various confounding factors like study design, duration, lifelong sauna habits, smoking, and physical activity, which could mediate the observed benefits [10]. Furthermore, there remains a critical need for standardization of sauna protocols across different populations and healthcare settings. Investigators should focus on developing individualized Sauna protocols based on patient risk factors and comorbidities, and develop a comprehensive protocol based on optimal temperature, duration of the session, and frequency to enhance both the safety and efficacy of sauna therapy in patients with different stages of IHD [59]. It is particularly important to consider patient-specific variables such as age, sex, baseline cardiovascular fitness, and concurrent medications when designing these individualized treatment regimens. Most studies of sauna treatment and its association with ischemic disease are observational studies with short-term follow-up. Additionally, many of these investigations lack adequate control groups and fail to account for potential selection bias, which may limit the generalizability of their findings. Future studies must monitor the longevity benefit and efficacy of Sauna over several years or decades to develop a deep and comprehensive understanding of the role of Sauna in both the prevention and management of IHD. Therefore, a large, well-designed, RCT is strongly recommended to evaluate the long-term relationship of sauna bathing in IHD [60]. These trials should incorporate standardized outcome measures, including cardiovascular mortality, morbidity, and quality of life assessments, to provide robust evidence for clinical practice guidelines. Also, investigators may dive into combining sauna treatment in conjunction with exercise and physical rehabilitation programs to enhance cardiovascular outcomes after IHD. Such integrative approaches may offer synergistic benefits and could potentially revolutionize cardiac rehabilitation strategies in the future.

## Conclusions

This review highlights sauna bathing, particularly Finnish and far-infrared types, as a valuable non-pharmacological adjunct for IHD, providing cardiovascular benefits such as vasodilation, improved endothelial function, enhanced nitric oxide bioavailability, reduced blood pressure, and decreased inflammation and oxidative stress. These effects resemble moderate physical activity, making sauna therapy especially suitable for individuals unable to exercise regularly due to physical limitations or comorbidities. Combining sauna therapy with conventional exercise-based cardiac rehabilitation may offer synergistic benefits through complementary mechanisms. Personalized protocols, considering genetic, clinical, and comorbidity factors, alongside digital health tools like wearable sensors and smart sauna systems, can optimize safety, monitoring, and efficacy. While generally safe for stable cardiac patients, unstable angina and recent myocardial infarction remain absolute contraindications. Future research should focus on long-term RCTs, standardized protocols, comparisons between sauna types and temperatures, and implementation strategies for technology-assisted therapy. Sauna therapy should be viewed as a supportive adjunct to standard medical care, integrated under proper supervision as part of comprehensive cardiovascular management.
